# Urban Emotion Sensing Beyond ‘Affective Capture’: Advancing Critical Interdisciplinary Methods

**DOI:** 10.3390/ijerph17239003

**Published:** 2020-12-03

**Authors:** Jessica Pykett, Benjamin W. Chrisinger, Kalliopi Kyriakou, Tess Osborne, Bernd Resch, Afroditi Stathi, Anna C. Whittaker

**Affiliations:** 1 School of Geography, Earth and Environmental Sciences, University of Birmingham, Edgbaston B15 2TT, UK; 2 Department of Social Policy and Intervention, University of Oxford, Oxford OX1 2ER, UK; benjamin.chrisinger@spi.ox.ac.uk; 3 Department of Public Health Sciences, Institute for Risk Assessment Sciences, Utrecht University, 3584 CM Utrecht, The Netherlands; k.kyriakou@uu.nl; 4 Department of Geoinformatics, University of Salzburg, 5020 Salzburg, Austria; bernd.resch@sbg.ac.at; 5 Population Research Centre, Faculty of Spatial Sciences, University of Groningen, 9700 Groningen, The Netherlands; t.osborne@rug.nl; 6 Center for Geographic Analysis, Harvard University, Cambridge, MA 02138, USA; 7 School of Sport, Exercise and Rehabilitation Sciences, University of Birmingham, Edgbaston B15 2TT, UK; a.stathi@bham.ac.uk; 8 Faculty of Health Sciences and Sport, University of Stirling, Stirling FK9 4LA, UK; a.c.whittaker@stir.ac.uk

**Keywords:** biosensing, mobile methods, urban wellbeing, interdisciplinarity

## Abstract

The use of mobile sensor methodologies in urban analytics to study ‘urban emotions’ is currently outpacing the science required to rigorously interpret the data generated. Interdisciplinary research on ‘urban stress’ could help inform urban wellbeing policies relating to healthier commuting and alleviation of work stress. The purpose of this paper is to address—through methodological experimentation—ethical, political and conceptual issues identified by critical social scientists with regards to emotion tracking, wearables and data analytics. We aim to encourage more dialogue between the critical approach and applied environmental health research. The definition of stress is not unambiguous or neutral and is mediated by the very technologies we use for research. We outline an integrative methodology in which we combine pilot field research using biosensing technologies, a novel method for identifying ‘moments of stress’ in a laboratory setting, psychometric surveys and narrative interviews on workplace and commuter stress in urban environments.

## 1. Introduction

The wide-spread availability of commercial and relatively affordable mobile biosensing technologies is shaping a new digitised and data-driven field of urban emotion analytics outside of laboratory contexts. The growth of the consumer market in connected wearable devices and smartphone use worldwide indicates an intensification of our apparently seamless, constant and intimate relationship with eHealth and digital technologies. Biosensing technologies which often collate a combination of physiological and psychological/survey data are now being used in the tracking of people’s emotional responses as they move through the urban environment, and in self-monitoring and management of emotional and mental distress.

The specific possibilities of researching emotional experience in cities through geospatial methods and location tracking has led to significant experimental work in this field, by geographic information scientists, environmental psychologists, neuroscientists and urban planners. These offer researchers exciting and experimental methods by which to ‘measure’ urban emotions, new data sources, and potentially novel relationships with research participants, for instance through citizen science methodologies [1]. Urban emotion sensing research often includes a combination of methods such as: (a) the collection of geo-located physiological or biometric data such as skin conductance levels or response or heart rate to indicate enervation of the autonomic nervous system, and thus stress [2,3,4,5,6]; (b) studies of cortisol levels in relation to urban or green spaces to indicate stress [7,8]; (c) neuroscience methods, such as electroencephalography (EEG) monitors to measure brain activity or reactivity [9] or post-hoc functional Magnetic Resonance Imaging (fMRI) [10]; (d) eDiaries, Ecological Momentary Assessment or spatially/biometrically/periodically prompted self-report surveys [3,4,10,11,12]; (e) interventions such as eMental Health smartphone applications [13].

Many of these mobile sensor technologies are proliferating in availability and use, despite a lack of research into their effectiveness, misgivings in terms of reliability, and unsettled questions about whether and how emotions can be measured. Researchers from both psychophysiological and humanities perspectives have expressed concerns about the direction of travel in the field of eHealth research. There are many confounding factors present outside of laboratory contexts which threaten the validity of research findings [14], and more fundamental issues of the interpretation of physiological responses being tracked within specific historical contexts [15,16,17].

In this paper, we argue that more attention needs to be paid to the significant epistemological and ethical problems associated with defining and researching urban stress, and—looking beyond issues of data privacy—to the ‘data practices’ and analytic convention of the emerging spatial science of urban emotions. These concerns are seldom addressed in biosensing studies on workplace and commuter wellbeing; research which is often has a remit of capturing and processing data on how and why people move through space and time in particular ways. To date, for instance, there has been a dominant research emphasis on reading off human stress responses to the physical, built environment. This is often achieved by splitting of objective and subjective emotions [6]. This has the effect of stripping human action from its social, political and historically situated environment, not least the nature of capitalist workplaces and the urban public sphere. It has thus been noted that a more specific account of space, time and context is necessary to understand urban wellbeing [18]. So too, the significant challenges of integrating objective and subjective data are often underplayed [19]. Whilst there is a thriving field of research on urban mobility and urban emotions (outlined below) this paper explores how paying more attention to data practices can advance interdisciplinary research on urban emotions.

Yet there remains much conceptual groundwork to be done in defining and understanding emotional dynamics in space. To this end, the paper pursues two main objectives. We use the conceptual tools of critical social theory to set out and compare a variety of disciplinary perspectives on the meaning of stress in relation to urban environments. This brings an interdisciplinary team of researchers together to integrate and analyse diverse forms of data, explicitly aiming to critically analyse our own data practices. The team included researchers from political geography, behavioural medicine, health psychology, GIS, urban planning and neuroscience. Researchers from political geography and GIS undertook data collection, and the other researchers provided perspectives on conceptual, definitional issues, discipline-specific methodological conventions and data analysis. Secondly, we introduce an interdisciplinary methodology, illustrated with a pilot study of the experiences of urban stress among people who work in urban environments to demonstrate the necessity of navigating between scientific and interpretivist modes of analysis to account for the complex relationship between the body, emotions and environment. It is increasingly commonplace to consider the emotional dimensions of urban mobility and transport planning through an attention to the sensory and affective experiences of commuters [20,21,22,23]. The intersection of senses, emotions and subjective wellbeing has shaped an extensive research field on both workplace and commuter wellbeing [24]. ‘Active’ commuting (e.g., by bike, walking) has been found to be less stressful than driving [25,26], longer commutes increase levels of stress [27], and the effects of a long commute impact on people’s mood at work [28]. More ‘ecological’ in situ studies which ask people to map their activity in real time, and rate their mood or positive/negative experiences of public space have also found that physical characteristics of cities such as distance from facilities can shape people’s subjective wellbeing as they move through space [18,23]. The appeal of mobile or wearable biosensor technology is that it promises to enhance these understandings through more ‘objective’ measurement of stress and wellbeing, which can be tracked by GPS in real-time [29] and can be integrated into smart city networks [30].

Similarly, in workplace and organisational management studies there is a long history of research on work stress and subjective wellbeing. This has found several drivers of work stress, including: physical surroundings of the workplace; workers’ control over their time; travel mode/duration; new technology; workload; performance pressures; role conflict; sense of responsibility; relationships with others; organisational culture [31]. Conversely, more autonomy and job control positively impact workers’ wellbeing [32]. In the rapidly developing field of affective organisational neuroscience studies, there have been concerted attempts to move beyond surveys of job satisfaction and subjective wellbeing, towards more biological forms of measurement, from monitoring employees’ hormonal levels [33], to efforts to detect frustration using EEG [34], and increasing attention [35]. These methods build on much earlier traditions of psychophysiological research in workplace settings, including a large number of studies on bus and lorry drivers, which brings together an interest in both mobility and work [36]. With novel enthusiasm for employee biosensing and emotion monitoring in the workplace the ways in which these technologies and the forms of data ownership and analysis associated with them become entangled in complex regimes of power, surveillance and control are rarely considered [37,38]. They themselves are part of the very working conditions and organisational climate which are known to shape work stress. We need to better understand how research methods and data practices contribute to these entanglements of power.

## 2. Methodological Approach

### 2.1. Epistemological Challenges of Defining and Measuring Urban Emotions

In this section, we use the method of immanent critique to de-centre the physiological account of the stress process which dominates the majority of experimental emotion biosensing intervention studies. Immanent critique, associated with Hegelian-Marxist philosophy and the Frankfurt School of critical theory [39], refers to a method of critical analysis which is “connected to relevant criteria and understandings internal to the culture or social order at which the criticism is directed” [40]. It is therefore a reflexive method which has a non-positivist epistemology. It encourages detailed descriptions, transparency and explicit statements of aims and biases. As such it does not attempt to eliminate bias in its methodology nor to generate data and evidence. Yet it is specifically interested in the experiences and drivers of diverse subjectivities and is therefore pertinent to understanding the social importance of research on urban stress. Its aims are explicitly normative; to highlight social tensions, contradictions, forms of oppression and relations of power. It is particularly relevant to a study of emotion biosensing precisely because it holds that scientific knowledge practices and technological developments are integral to the feedback loops through which we come to know ourselves through subjective experience.

To begin with scientific knowledge practices; In the physiological account, stressors are seen to arouse an autonomic nervous system (ANS) which controls our internal organs and maintains our homoeostasis or biological balance [41]. The arousal of the sympathetic nervous system can be identified using measures of blood pressure, heart rate, hormonal secretion, electrodermal activity (sometimes referred to as skin conductance), and respiratory activity. However, it should be noted that components of this system are also activated through other activities besides stress, such as physical exertion. This makes it difficult to distinguish stress-related arousal from physical arousal, meaning that interpretation of so-called ‘objective’ measurements must necessarily involve subjective analysis. Further, stress is also a highly ambiguous term, which can refer to the stressor (input), a physiological or psychological processing system, or the stress response/output (neural, emotional, behavioural, biological) [42]. In evolutionary theory, stress motivates people to act, and stress response is a necessary prerequisite for survival and adaptation to our changing environment. Psychological research on stress is similarly multi-faceted. It can, for instance, focus on the cognitive processing or appraisal of stress, the mediation of stress response by personality type, or one’s capacity to cope in a given situation.

Influenced by the philosophical (hermeneutic) traditions associated with immanent critique, there is an emerging acknowledgement in ‘biosocial’ research on urban stress that stress cannot be easily delineated and measured [43,44]. The biosocial approach is concerned with the ways in which human subjectivity and action are always already shaped by the knowledge practices of the biological sciences. It also pays attention to structural determinants, cultural and political processes, and risk factors which shape our vulnerability to and/or specific manifestations of stress in specific environments or socio-economic contexts [45]. Therefore, stress can operate at a societal level as much as through the body’s physiology—more broadly, feelings are embodied, but can be “locked into place by social, relational and material circumstances” [46]. Historical accounts of stress have shown how stress measures were originally developed in a military and industrial to improve task performance of soldiers and workers in post-war North America [47]. This highlights how stress is used both as a universal biomedical term, and is culturally produced in a specific historical context; the “pathways to embodiment” are “eco-social”; they, include socially constructed meanings, historical formations, technological mediations and epidemiological dimensions, and operate across many scales of analysis [48].

There is a sense that human emotions can be measured and understood in much the same way as an ecology of animal behaviour. A wide range of important critical social science analyses challenge this notion. To take the complex and situated history of the concept of stress into account, researchers need to consider how their methods make certain claims to know and represent embodied urban stress. In this sense, technological developments are never neutral, and data assumptions and practices need to be considered. For instance, the distinction between subjective and objective forms of data is increasingly being problematised in urban emotion research. Previous attempts to establish new interdisciplinary foundations for research on body-brain-environment interactions include Christian Nold’s [49] artistic projects on ‘emotional cartographies’/‘bio mapping’. Nold uses GPS and a galvanic skin response (GSR, also called electrodermal activity) sensor worn on the finger to indicate the physiological arousal of participants as they walk through urban space. This is followed by participatory workshops which involve discussion of the mapped data using Google Earth. He describes how during these workshops the participants: “…*were not narrating by themselves but were performing together with the spikes of the arousal data to make sense of what they had seen, heard and felt on their journey and to share it with the group*”.

This idea of ‘performative visualisation’; i.e., the construction of representations through human, technology and social interaction, is a means to elicit new narratives, and to articulate in its complexity and potential contradiction the relationship between emotions, body and environment. This produces new insight from the reflexive perspective of research participants in socially interactive situations and new forms of data integration should keep tensions, contradictions and inconsistencies in play. For Nold [49], the ethical dimension here is to bring urban emotion into the realm of collective concern through a participatory approach to GIS research, of which there is a substantial literature.

As such, Nold provides new ways of talking about the body in the context of ‘posthuman’ agency. This is not a return to an animal model of human behaviour but a recognition of the ways in which our emotional responses to space are always already technologically and socially mediated. It denotes a second important epistemological challenge for urban emotion research: finding ways to acknowledge the already significant adoption and prevalence of self-tracking technologies in contemporary social life, particularly in the post-industrial urban context in which much of this research has been conducted. There is a double hermeneutic at play in urban emotion research, since human subjectivity is embedded in technological and material lifeworlds rather than separate from them.

One dimension of this problem is the need for analytical approaches which can switch between paradigms, and productively engage with (rather than attempt to resolve) the deep-rooted historical conflict between positivist and interpretivist forms of analysis. This requires forms of ‘interdisciplinary entanglement’ which are based on a level of shared understanding which is sometimes not particularly forthcoming, particularly when it comes to brain-body-environment relationships [45,50]. Post-phenomenological research on urban mobility [51], and the combination of Interpretivist Phenomenological Analysis and biometric research on stress in green spaces [7] offer exemplars which bring together sensory experience with the personal narratives and judgments of participants in given situations. Similarly, recent research on commuter stress in human geography approaches habitual forms of embodied stress “in terms of the changing capacities for affecting and being affected that take place through time, rather than a personal, psychological and predictable response of fully formed individual bodies” [20].

A third challenge refers to the specific spatial and-temporal imaginaries assumed in experimental urban emotion sensing research. Because of necessary reductionism involved in the design of new biosensing technologies and rendering emotions measurable, it is important to admit that something is lost in the equation of ‘space’ with GPS location, mapped physiological emotional data and geo-located subjective reports or sentiment analysis at specific and fleeting points in time. Firstly, the idea of ‘context’ has been reduced to objects which are within a person’s (often visual, sometimes auditory) perceptual range, or aspects of the physical built environment which may act as emotional triggers (e.g., disrepair, crowds, traffic). Human geographers have long dismissed the conceptualisation of space as a mere container for human action. To do so also omits consideration of the urban environment as a public sphere, a space of social encounter, and attention to emotional factors (e.g., memory, reflection, appraisal, judgement) which may be personally specific, socially produced, and develop over much longer timescales.

This has been partly addressed in Helbich’s research on environmental exposures and mental health outcomes [52]. By adopting a theoretical approach which accommodates both people’s daily mobility through space and their movements throughout the life course, this work expands our spatial thinking on urban emotions beyond the immediate environment, and pays close attention to temporalities such as duration, sequence and accumulation of epidemiological exposure to mental health risk factors. In this case, the focus is on depression, suicide and emotional responses commonly deemed pathological. In our study, however, we wanted to go beyond a medicalised account of mental health to recognise that the measurement of situation-specific emotions, such as stress, has a particular history, and historical causes. We therefore used a mixed-method study design including biosensing, Ecological Momentary Assessment, stress and wellbeing surveys, and qualitative interviews. These directly investigated the meanings and experiences of ‘urban stress’ as articulated by our research participants in the particular context of their experiences of stress both getting to and moving within the workplace.

### 2.2. Location

The study was conducted between November 2017 and January 2018 in two contrasting cities: Birmingham (UK) and Salzburg (Austria), selected to reflect a stark diversity of urban environments. It was part of two larger projects, one which explores urban wellbeing in different national contexts and another which is developing novel geospatial methods for measuring urban emotions. Birmingham is the UK’s second largest city, with a population of c1.1 million. This post-industrial Midlands city is therefore substantially larger and more densely inhabited than the archiepiscopal, baroque Alpine city of Salzburg, which has a population of around 150 k. It is not our intention here to provide a comparative account of urban wellbeing in these two distinct urban environments, though it is worth drawing attention to the often underacknowledged heterogeneity of the category of the ‘urban’.

### 2.3. Study Participants

The eligibility criteria for participants were that they were adult volunteers who were currently in work in one of the case study cities. In total, 31 participants were recruited through one Higher Education Institution (HEI) workplace in each city (see Table 1). Participants were recruited via ‘Workplace Wellbeing’ staff, posters and flyers distributed at a Workplace Wellbeing event, a union mailing list, and in the case of Salzburg, through personal contact with HEI staff. Our non-representative and self-selecting target sample included both men and women, a range of ages between 18 and 70 to reflect the working age population, and a variety of job roles including both academic and non-academic staff. There was an imbalance of genders and commute modes and mean ages in our sample, but our aim was not to provide a statistical comparison between the two cities.

### 2.4. Ethical Approval Process

Participants were provided with detailed information about the study aims, methods and how data would be collected, managed and shared back with participants. They were asked to opt-in to the study voluntarily through providing written informed consent. Data was anonymised through the use of pseudonyms. Participants were able to take pauses during the interviews and withdraw from the study up to 4 weeks after data collection, and we were informed by discussions of an ‘ethic of care’ within mobile methodologies in health and wellbeing research [53]. Ethical approval was granted by the first author’s institutional ethics board (November 2017: ERN_17-1224 09).

### 2.5. Data Collection

#### 2.5.1. Biosensing

We used a wrist-worn medical grade biosensing device (Empatica E4, Empatica Inc., Cambridge, MA, USA) to collect time-stamped biometric data continuously (at a frequency of four samples per second) throughout the participants’ journeys to work, their working day and their journey home on one day. A detailed instruction sheet was provided to explain how to use the device. We asked participants to wear the sensor for 5 min sitting still at home in order to obtain baseline measures. Participants were also asked to manually tag an event on the device when they arrived at and left work using one simple button, so that we could isolate their journey from the working day. We collected biometric data including changes in electrodermal activity (EDA), blood volume pulse (BVP), wrist movement (tri-axial accelerometry, showing the three-dimensional movement of participants), heart rate (HR) and skin temperature.

#### 2.5.2. Ecological Momentary Assessment (EMA) Diary

Participants were asked to complete a diary at hourly intervals over one day. This noted any food and drink intake, their main activity, and asked them to respond to the following questions: (1) What are you doing right now? (free text) (2) What is the main feeling you are currently experiencing? (3) What is the intensity of this feeling? (15 options: alert, excited, elated, happy, bored, depressed, sad, contented, serene, relaxed, calm, upset, stressed, nervous, tense), on a 3 point scale: extremely, very, quite) (4) How stressed are you feeling right now? (5-point scale from extremely to not at all) (5) How do you rate your ability to cope with this situation? (5-point scale from very poor to very good). The purpose of this was to be able to combine the objective biometric data with subjective momentary self-report of participants’ own stress and wellbeing. In Salzburg we trialed an eDiary which prompted responses to these questions at random times but replaced this with a paper diary in Birmingham due to problems of internet connectivity.

#### 2.5.3. Stress and Wellbeing Surveys

We asked participants to complete three surveys at the end of the day, on completion of the biosensing and EMA diary data collection. The first recorded general demographic data including gender, age, education level, marital status, perceived health status, employment role and duration, job satisfaction, mode and length of commute to work. The second was the 26-item World Health Organisation Quality of Life Survey (WHOQOL BREF [54]) which covers aspects of physical health, psychological wellbeing, social relationships and environment. Responses are measured on a 5-point scale from very poor/not at all to very good/completely and so on. The third survey was the Perceived Stress Scale-10 (PSS [55]) which asks participants about their own perceptions of stress (e.g., feeling upset, nervous and stressed, coping with difficulties and feeling in control). Both are retrospective surveys, requiring a degree of self-evaluation and reflection of the previous two weeks (WHOQOL) and one month (PSS) respectively.

#### 2.5.4. Qualitative Interviews

In order to provide an interpretive and narrative account of participants’ experiences, two members of the research team with extensive qualitative interviewing experience conducted semi-structured qualitative interviews, in English, with each participant (lasting between 30–60 min), usually the day after biosensing data collection (in two cases this took place a few days later). These interviews followed a topic guide which including participants’ feelings about their journey to work, their personal circumstances and background across different timescales, their own conceptions, definitions and experiences of stress and its relation to bodily feelings, urban environments and societal change, and their expectations and experiences of involvement in the study and measures used. Participants were sent copies of the participant information sheet before the interview. All 31 interviews were audio recorded and transcribed verbatim and thematically analysed.

### 2.6. Data Analysis Methods

#### 2.6.1. Biosensing Data—Developing an Algorithm to Identify ‘Moments of Stress’

In order to analyse the biosensing data, we developed a rule-based algorithm to detect “moments of stress” (MOS) using physiological signals (electrodermal response and skin temperature), and combining the rule set empirical findings with input from psychologists’ and medical researchers [56]. They confirmed that the chosen physiological parameters are sensible from a psychophysiological and emotion psychological perspective, according with a review of Autonomic Nervous System activity in emotions [57]. The measurements were collected in a laboratory setting with the Empatica E4 and BioHarness (Zephyr, Boulder, CO, USA, a chest belt measuring a variety of cardiological parameters) selected after technology evaluation and laboratory tests [58].

Our method extracts MOS from the measured data using a customised signal analysis procedure. First, a low-pass filter (cut-off frequency 0.5 Hz) eliminates high-frequency variations in measurements that may be caused by technical inaccuracies, followed by a high-pass filter (cut-off frequency 0.05 Hz) to filter the tonic GSR as an indicator of each participant’s baseline. Second, a down-sampling process is applied (from 4.0 Hz to 1.0 Hz) to obtain one value per second establishing comparability between the signals in the frequency domain. Then, our rule-based algorithm detects patterns in the measurement data that are taken to indicate a physical stress reaction: 5 s of GSR increase, followed by a temporally delayed (+3 s) decrease in skin temperature. Next, the algorithm looks for a local maximum followed by a local minimum in skin temperature together with a slope steepness in the GSR increase of ≥10°.

The required calibrated data for the algorithm were obtained through a preceding laboratory experiment that we carried out. We induced auditory stimuli while the physiological signals of participants were recorded with the abovementioned wearable sensors aiming to connect physiological responses to a stressor. The constrained environment of laboratory experiments permits eliciting, controlling, and measuring an emotional response reliably [14]. We also asked the participants to note down their perceived stress intensity after each stimulus using a 5-point scale. We then gathered field data including the present study to evaluate the efficiency of our algorithm during real-life events recruiting both cyclists and pedestrians in various cities. The global average accuracy of detected MOS was 84%, and the congruence amongst detected stress, self-reported stress (eDiary entries and interviews) and recorded stress (first person video) was high (see [56] for full details).

#### 2.6.2. EMA Diary and Surveys—Descriptive Statistical Analysis

The EMA diary, PSS and WHOQOL-BREF survey data were compiled into an Excel spreadsheet, to enable them to be integrated with our basic descriptive summaries of the interview data, and biosensing data. We used this spreadsheet to record where each data type indicated a binary categorisation of feeling stressed or not stressed in order to examine how consistent these data types were in indicating moments of stress. We categorised the PSS data in terms of participants having PSS above or below the average rating of 13 [55], and the WHOQOL in relation to the population norm score of 72.6 [59]. We compared the EMA Diary and survey results with variables such as commute duration and commute type, using various statistical tests (ANOVA, F-test, Levene’s test and Tukey test) in order to assess these relationships. These Open Access data are available in the Dataverse repository (https://doi.org/10.7910/DVN/D2FOTT).

#### 2.6.3. Qualitative Interviews—Thematic Analysis

Inductive thematic coding of the qualitative interview data was undertaken by the first author, according to a coding framework emerging from the interview data (see Section 3.2), using NVivo 12 qualitative data analysis software (QSR International)). Thematic codes were sub-divided into more specific sub-codes in an iterative manner, based on the principles of grounded theory. The grounded theory methodology holds that theories emerge in the conduct of research, through close and systematic reading of interview data alongside engagement with existing theories. Themes are developed through the active interpretive strategies of the researcher; the data cannot be said to ‘speak for itself’ [60].

### 2.7. Data Integration

**Figure 1:**
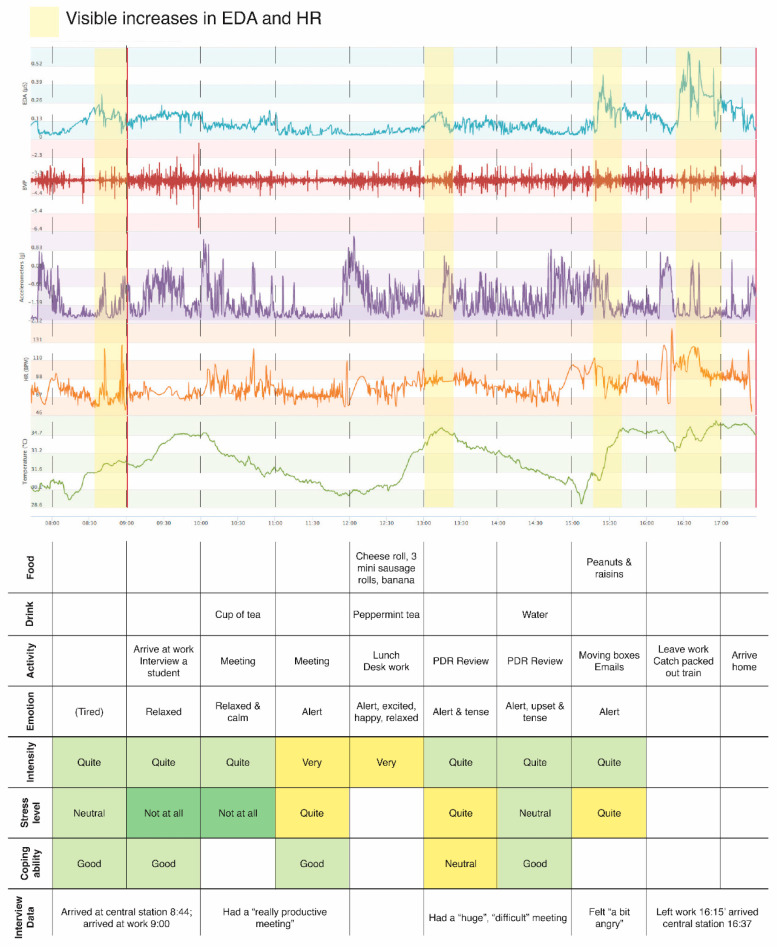
Example of biosensing and diary data visualization—feedback sheet for participants.

One of the key challenges identified above is in how to successfully integrate different forms of data in ways which can go beyond mixed methods approaches to offer more genuinely interdisciplinary advancement. We compiled all the data sources together to analyse the relationships between the different conceptualisations of stress: biodata (physiological), surveys (psychological), and interviews (experiential). A key priority was to be able to provide research participants with an interesting visualisation of their own biodata, integrated with the survey and EMA diary data (see Figure 1 for an illustrative example). Participants were also provided with their interview transcripts. We would argue that taking an ethical approach would necessitate the further extension of this participant involvement into the kinds of performative visualisation and community mapping described by Nold [49]. This would make specific embodied forms of data more visible to participants and researchers alike, elicit additional qualitative responses which could democratise the practices of data analysis and interpretation. For example, critical/feminist GIS researchers have advanced iterative, reflexive and exploratory techniques of “grounded visualisation” [61]. This method has been explicitly deployed in a biosensing study of urban heritage environments in the UK [62]. The approach allows for attention to both the general and the particular, often involving participants in the practice of coding data into general categories, whilst maintaining a sensitivity to the partiality and positionality of knowledge.

## 3. Results

Knowing that we would have the small sample size commonplace in experimental biosensing research, we did not set out to test a specific hypothesis about the differences between these cities, or to unambiguously identify the drivers of workplace or commuter stress, but to provide proof of concept for the forms of data analysis that would be possible. In doing so, the aim was to explore and test the theoretical and methodological constraints and opportunities of an interdisciplinary approach to urban emotion sensing. In this spirit, this section describes the main findings relating to broad and indicative research questions which would be of interest to researchers of workplace or commuter stress, such as how commute duration or type would influence stress, differences between stress at work and during journeys. By investigating the correlation and relationship between the different data sources, we consider the validity of the different measures.

### 3.1. Psychometric Stress, Wellbeing and Commute Duration/Type

We recruited 22 participants in Birmingham (19 females; 3 males) and 9 in Salzburg (1 female; 8 males). The mean age of participants was 39.4 (SD = 11.7). A high proportion of the sample reported they were satisfied or very satisfied with their job, and many participants had been in their current job for over 2 years. With reference to the psychometric measures of stress and wellbeing used, participants in Birmingham had higher average perceived stress score (17.1; SD = 5.0), above the average of 13. For Salzburg this was below the average (11.5; SD = 5.7). The mean WHOQOL score was 73.8 (SD = 7.9), with a higher quality of life score was found in Salzburg (79.0; SD = 7.5) than Birmingham (71.8; SD = 7.3) (see Table 2). Both were above the population norm WHOQOL score of 72.6 [59].

Most participants had a commute time of 15–45 min (74%), and seven participants had a commute time of over 45 min (two reported their journey took over 2 h). 45% travelled by car (but none in Salzburg), 32% by train or bus, and 19% (six people) cycled or walked.

Through a one-way ANOVA, we found that the commute durations significantly differed on PSS score (F(4,23) = 3.40, *p* = 0.02). Concerning the commuting type, Table 3 presents the respective descriptive statistics revealing that people who use bikes to commute have the lowest mean PSS value. On the contrary, car drivers have the highest mean PSS value followed by participants riding the bus.

We further used the ANOVA F-test method to determine whether the mean PSS score for each commuting type was statistically different, as Levene’s Test for Equality of Variances showed no significant differences (F(4,23) = 0.77, *p* = 0.553). The F-test revealed that the means are statistically different for a confidence level of 95% (F(4,23) = 3.33, *p* = 0.03). To explore which means of commuting type are statistically unequal we used the Tukey post-hoc test that detects differences through a pairwise comparison. For a confidence level of 95% the multiple comparisons showed that the mean PSS score statistically differed only between car drivers and cyclists (diff = 9.5, *p* = 0.03).

There were no significant differences on the WHOQOL score by commute duration (F(4,23) = 2.80, *p* = 0.14) or type of commute (F(4,23) = 2.86, *p* = 0.19). Table 4 depicts the descriptive statistics for WHOQOL and commute type. Cyclists have the highest mean WHOQOL value (m = 83). On the contrary, people who commute by car or bus exhibit the lowest mean WHOQOL values (m = 69.23 and m = 69.75 respectively). Levene’s test of variance led to the result that there is not significant difference between the commuting types on WHOQOL scores (F(4,23) = 2.39, *p* = 0.1798). The F-test indicated a statistical difference, but the *p*-value was 0.047, which introduces ambiguities due to the closeness to 0.05 for a confidence level of 95% (F(4,23) = 2.86, *p* = 0.047). The multiple comparisons through the Tukey test revealed a statistical difference for the mean WHOQOL values between car drivers (diff = 13.25, *p* = 0.04) and cyclists for the same confidence level. The same is true for the relation between PSS and commuting type.

### 3.2. Spatial and Temporal Differences in Urban Stress Experiences

**Figure 2:**
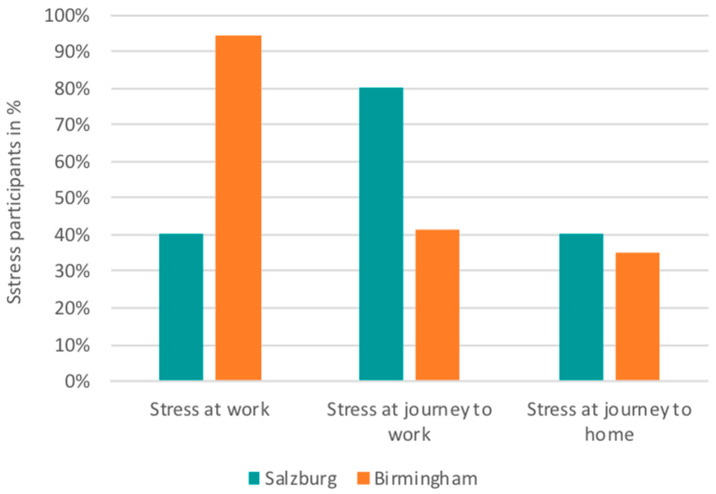
Percentage of participants characterized as “stressed” based on physiological measurements indicating Moments of Stress.

We used the developed algorithm to detect MOS of participants in Birmingham and Salzburg during the journey to work, the journey back to home and during the working hours. Figure 2 depicts the descriptive differences between the two cities. MOS were observed in the physiological data for over 90% of the participants at work in Birmingham. The corresponding percentage for Salzburg is significantly lower, as 40% of the participants experienced MOS. Concerning urban commuting, the analysis of biodata reveals a different insight for stress responses. More participants in Salzburg had MOS during trips than in Birmingham. More specifically, 80% of the participants in Salzburg had MOS during their trip to work and 40% in Birmingham. The journey back to home had fewer MOS (compared to the other two periods) in the two cities, and the percentages are considerably decreased mainly for Salzburg (~40%) and slightly for Birmingham (~35%).

**Figure 3:**
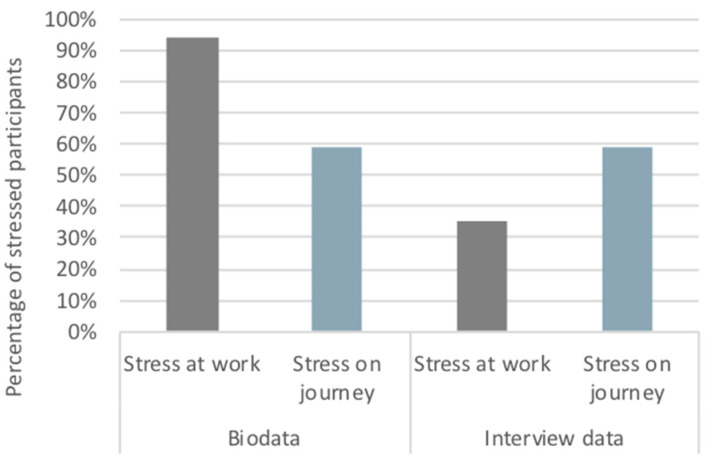
Percentage of participants characterized as “stressed” during journeys and working hours in Birmingham: Physiological measurements (biodata) vs. Interview data.

Figure 3 presents the comparative analysis between measured (physiological data) and reported stressful states at the workplace and in urban environments during the commute in Birmingham. We categorised “stressed participants” as those who experienced at least one MOS per period, and we acknowledge the necessarily contested nature of doing so. Interview data correspond to physiological data concerning the articulation of stressful responses during the trips as the percentages are equal. However, our data would seem to suggest that work triggers more stressful states than participants perceive. The number of stressed participants is considerably higher based on physiological measurements exceeding the percentage of 90%. On the contrary, only 35% of participants reported stress during working hours at the interviews. Participants’ mood or social desirability bias may affect psychometric responses during the interview, and this could be a reason for the significant discordance between the physiological and subjective accounts. Clearly then, we have already highlighted the non-equivalence of objective and subjective stress, which points to the need for novel forms of data integration and analysis in studies which aim to combine these.

**Figure 4:**
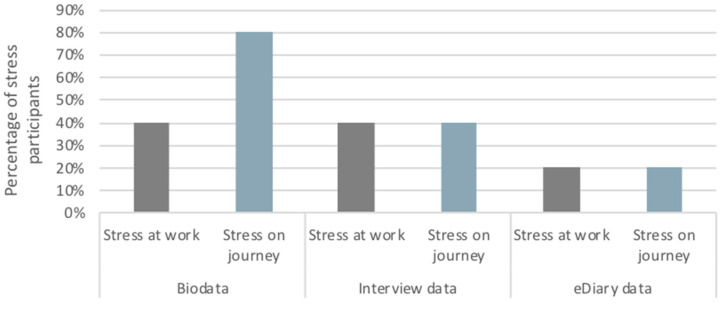
Percentage of participants characterized as “stressed” during journeys and working hours in Salzburg: Physiological measurements (biodata) vs. Interview data vs. eDiary entries.

During the field test in Salzburg, the participants also had the opportunity to report their emotional states using the mobile electronic version of the EMA Diary (eDiary) application (see [4] for technical detail). Figure 4 presents the rates of stressed participants based on the physiological measurements, the interview data and the eDiary entries. First of all, we notice the discrepancy between interview data and eDiary entries. Both of them are subjective inputs and represent the (cognitively) perceived emotional states. However, the analysis of the interview data revealed higher percentages of stressed participants. This could be explained by the fact that eDiary application requires interaction with mobile phones to record the emotional states, which may be challenging during commuting especially for cyclists.

According to eDiary entries, both work and journeys triggered stressful responses only at 20% of the participants. Interview data disclose a higher percentage of stressed participants (40%) for both work and trips. The physiological data picture a different situation for participants’ emotional states. In this case, the percentage of stressed participants during the journeys is much higher (80%) than the respective data from the interviews (40%) and eDiary entries (20%). On the contrary, interview data are in line with physiological data for the case of stressful responses during working hours. So, this phenomenon raises the question of whether the participants are actually stressed during urban commuting, since the repetition of the same triggers may have led to habituation. Otherwise, we could tentatively conclude that urban commuting elicits more stressful responses than work among the Salzburg participants, notwithstanding the ‘active’ travel modes that most of them were engaged in (including no car users). Further analysis of the effect of mode of travel with a larger sample size would through light on this issue.

The discrepancies amongst the different methods of collecting emotional data allow us to shape different outlooks for urban systems and the associated emotions in the studied cities (see Table 5). While we make no assertions of conclusive evidence, at a merely descriptive analytical level, we note that the biodata portray Birmingham as a city where work provokes more stress than urban commuting, while perceived emotions reveal the opposite. For Salzburg, subjective measurements present urban commuting and work as equally low stressful tasks. The biodata also showed that commuting is significantly more stressful than work. This could be associated with the finding that commute duration is the only parameter amongst age, marital status, job satisfaction and education level, that is correlated with PSS for Salzburg at a confidence level of 95%. For Birmingham, none of the aforementioned parameters is correlated with PSS for the same confidence level. Larger sample sizes and a protocol based on specified routes determined by the researcher in each city, could be used to develop a methodology for robust comparison between such diverse urban systems.

### 3.3. Experiences of Embodied Stress

The table above provides selected examples of some of the most frequently used codes from the interviews to show the kind of topics and perspectives which were covered, and we elaborate below on a selection of main including: participant reflections on the experimental study and measurement; experiences of self-tracking, narratives of the journey description, and participant interpretations of the causes of stress.

#### 3.3.1. Participant Reflections on the Experimental Study and Measurement

Previous research on the psychophysiology of stress has shown that people’s bodily responses don’t always correspond with their psychological state, even if people strongly expect them to [63]. People can variously become habituated to or sensitive to stress response, and their experiences and expressions of emotions can be diverse and complex. This was recognised by our research participants. Some expressed concern that our measures should simultaneously reflect their embodied states, their reported feelings (via survey) and their reflections on these in an interview situation:

*“I don’t know if you want to look at the wording. Because sometimes—*[long pause]* I know I’m going, and I’ve got adrenaline going, and the results might look like oh you look stressed because your readings are—so I put it I’m not necessarily stressed, as in er, er, er. Stress as in—I’m not stressed, I’m* [long pause] *alert. And I’ll tick that box at the time*.” (Birmingham Participant 10, female, age 30–34).

“*There were times when I was, like, oh I’m a bit tired now but there wasn’t an option of ‘tired’ and there was a time when I was a bit annoyed and there wasn’t, you know, ‘irritated’ or ‘annoyed’ or that kind of thing, but I suppose it’s very difficult to narrow down exactly what words you’re going to put on there.*” (Birmingham Participant 3, female, age 35–40).

“*My idea of stress is the sort of feelings that you get where everything’s getting at you and you’re just getting more—for me I get wound up, I get like angry. Stress is always when people are getting pushed at work where they can’t cope. Stress is something that affects how you make decisions and work in a sort of irrational, makes people act in an irrational method, irrational manner because of the emotions they start feelings because of the external factors, that someone is pushing onto them, that they can’t control or can’t alleviate*.” (Birmingham Participant 17, male, age 25–30).

These questions of subjective meaning challenge the notion in both emotion biosensing and critical biosocial research that people generally see themselves through a biological lens. A study found that “people do believe biosensors can reveal their thoughts and feelings” [64]. This echoes the contention of sociologist Nikolas Rose whose work has been central to advancing the biosocial research agenda, that the data practices and imperatives of the new bioeconomy have given rise to “a new ‘somatic’ sense of ourselves, which extends to self and identity itself” [65]. Rather, our findings suggest that there is an ongoing tension and negotiation of what participants expect biosensing technologies to be able to measure, how they feel and how they can describe or speak about their stress both as an emotional construct and an embodied experience. This reflects what biomedical anthropologists and sociologists have described as the complex relationship between scientific knowledge practices, laboratory and visualisation technologies in producing ‘subjectivity’, how we think about “what makes us who we are” [66].

#### 3.3.2. Self-Tracking

There are looping effects which we must take into account when attempting to integrate objective and subjective data [66]. So often, no articulation of the process of subject formation (and thus subjective wellbeing) is offered where stress responses are inferred from data on the built environment. Yet research participants themselves do not ignore this circularity or looping of social and embodied experience. For instance, some participants, shaped by a technological environment already saturated with demands to ‘know’ one’s one emotional state would have preferred the opportunity for a more basic representation and ‘capture’ of their feelings:

Participant: *Yeah, well they’re all funny emotions really because they remind me a little bit of like Facebook ‘feeling whatever’* -

Interviewer: *Do you think little emojis would have been better*?

Participant: *Yes, probably.* (Birmingham Participant 12, female, age 45–50)

But others were more critical of the influence of the technological environment on societal levels of stress and our experience of time:

“…*we’re kind of chasing our own tails now. Because you can do everything instantly. Which is great, on the doing end. But on the receiving end, and the feedback end, they want that instantly as well. So we’re doing things faster, and making ourselves do things faster. So we’re not—we haven’t really won much have we?*” (Birmingham Participant 10, female, age 30–34).

In this account, it is clear that stress is not primarily considered through a physiological perspective, but refers to a set of cultural and social norms about how we relate temporally to the world, including what constitutes a successful life in this context.

#### 3.3.3. Journey Description

In this respect, the variety of methods we deployed were helpful in terms of looking across different spatialities and temporalities of analysis. In terms of commuting, several participants highlighted potential environmental stressors on their journeys to and from work, including (for car users): roadworks; icy conditions; particularly difficult junctions; lack of car parking; (for rail/bus users): overcrowding; lack of seating; late running or cancelled services; noise. Cyclists too expressed that they were stressed where there were difficult junctions or aggressive car drivers. A common theme was that other people’s behaviour or presence was the most common cause of commuter stress. But commuter stress was also mediated by sources of stress which were societal, rather than environmental—linked to circumstances which were not directly observable or responsive to stimuli in the urban environment.

For instance, participants described how their commute stress was induced by the fear of reprisals for lateness by their workplace manager, or by their views about how much governments should invest in transport infrastructure. By contrast, a pleasant or ideal work commute for many participants would involve using the time to speak or correspond with family and friends, undertake another activity such as reading or work, or provide socially-valued opportunities for fitness and health (cycling/walking). This confirms research which has explored factors which mediate commuter stress, such as productive use of planning time and ability to plan travel [67].

#### 3.3.4. Causes of Stress

Attributions of the causes of stress were for many participants related to power relations associated with gender or autonomy at work [68]. For instance, some of our female participants described the domestic pressures they felt and the compromises they made in terms of spending time with their families, stating a lack of autonomy and a sense of ‘prescribed time’ as a key determinant of stress:

*“…there is a constant stress because of the ability to be an adequate mother to my daughter and the time—so in the week there’s very limited time, you know, it’s a case of, it’s very prescriptive.*” (Birmingham Participant 16, female, age 35–44.)

In some cases, what was causing stress was not restricted to a specific space or time, but a person’s interpretations of the wider socio-economic environment, which were no less real than their physiological responses:

*“There seems to be a shift from people being a lot more relaxed to more stressed. Almost like people have become more stressed about money and also with the uncertainties that have been going on in politics at the moment it has increased people’s stress levels because are unsure about jobs, food, fuel and all these things that people don’t have control over.*” (Birmingham Participant 14, female, age 50–54.)

*“I think people—I mean, things like food banks. People are going to go hungry again, which I can’t remember, I don’t think people did really in my lifetime, but in my parents’ lifetime they could remember when people went hungry. I think a lot of people are going to find their life is very much worsened. And I think that’s going to impact on my life because I’m going to notice that. I don’t like seeing a lot of poor people. I don’t like seeing people having what is obviously a poor quality of life. I find it very dep-, I thought that when I was a child and we used to go in from the suburbs where we lived, through the inner-city area in Manchester, and you could see people who were obviously poor. So I do find that depressing and it does impact—and stressful. It makes me worry for the future*” (Birmingham Participant 5, female, age 65–70).

In this sense, the experience of stress can be highly contingent on perceptions of what are clearly some quite subjective, political dimensions of the state of society and one’s place within it. By undertaking more participatory forms of research such as performative visualisation and collective engagement with the biosensing data, for instance on a study which focused on one element of commute or work stress (e.g., autonomy, social relationships, or political climate), we could envisage how mobile sensor methods could enable participants to bring into view both their embodied reactions to stress and their narrative reflections and judgements on the reasons and solutions to this. This could inform workplace wellbeing initiatives by voicing collective concerns and organisational cultures, using data which demonstrate the physiological expression of stress as a starting point.

#### 3.3.5. Limitations

Although the aim of our research was not to compare the physiological differences in work and commuter stress between the two contrasting urban areas, our methodology has the potential to move closer towards this. In other words, with a much larger sample size and more a controlled experimental protocol (e.g., pre-planned, commensurate walking, cycling, train or car journeys of equivalent lengths, times of day, participants of the same age and physical mobility, socio-economic status, measures to avoid self-selection bias and a more comparable sample in terms of gender, mode of travel), it would in principle be possible to develop robust evaluations of the liveability of particular urban environments in terms of their impact on emotional wellbeing. This possibility is enhanced by the integration of methods that we piloted in this study, and is noted as a valid approach for urban environmental health research using geolocated N-of-1 datasets [69]. The MOS algorithm also has some limitations which support the need for mixed methods approaches and the advancement of standards of data integration and triangulation in mobile research. Physical exertion as research participants move through space modulates the activity of the autonomic nervous system (ANS) as it activates the same physiological processes as responses to emotion psychological processes. Incorporating the accelerometry data from the E4 biosensor could potentially be used to address this in future studies. An additional significant aspect is that the algorithm provides a binary classification of stress (stress or not) which does not take into account the multi-disciplinary perspectives on stress that we want to highlight in this study. People may experience stress at multiple strengths [70] so the algorithm may not be able to detect low-intensity MOS. Additional methods are therefore required to recognise stress both as a continuum and as a situated and adaptive emotional response, though this simple measure may help future researchers begin to interpret the relatively large quantities of data generated with biosensors. The use of the diary was a further limitation, both in the sense of its perceived intrusiveness in participants’ daily routines, the relatively short duration of diary use (1 day), and its lack of sensitivity to more long term of chronic forms of stress. The integration of qualitative interview data poses a limitation in terms of ‘muddying’ the definitional boundaries of stress, emotions, perceptions of urban environments and experiences of embodiment. But on the other hand, this also allows us as researchers to acknowledge some of the personal, social and cultural complexity of urban stress and the heterogeneity of people’s different reactions, urban experiences and engagement with what are non-invasive, non-laboratory research methodologies but which nonetheless intervene in people’s ordinary relationships to urban space, other people, their work and commute environments, wearable technology and their own emotions. Despite these limitations, the results from the real-world field-studies indicated that real-world situations elicit sufficient variance in the stress response to meaningfully detect MOS.

## 4. Discussion

Our pilot study found basic differences between commute duration, type and participants’ perceived levels of stress, as measured by the psychometric Perceived Stress Scale, but not the WHO Quality of Life wellbeing survey. This confirms findings from existing research, but the non-equivalence between both survey responses suggests the need for researchers to be tentative in the conclusions that can be drawn regarding the nature of stress or wellbeing that is being measured. Similarly, we showed discrepancies between the outcome measures informed by varying definitions of stress (MOS—psychophysiological; interviews—qualitative subjective experience; PSS/WHOQOL/eDiary—quantified subjective scales). This indicates that there are important distinctions between ‘mood’, momentary affects, long-term strains and health, quality of life or wellbeing impacts of stress. These are increasingly recognised in the work/commuter stress literature [24] and should be explicitly and consistently drawn out. Care must be taken when reporting findings about the causal pathways and personal impacts on the multi-faceted concept of ‘stress’, but this also opens up opportunities for researching temporally-specific environmental triggers of physiological stress indicators. For instance, using our MOS algorithm, we also found that journeys to work were more stressful than journeys home. This method and measure could be usefully applied to investigate the psychological processes of anticipation in commuter stress [71].

Through our qualitative interviews, the study was able to explore wider time-spaces of stress in relation to personal experience, with the aim of avoiding the reduction of stress as a response to environmental triggers. These conversations demonstrated how psychophysiological responses are mediated both by the science of stress and by our intimate relationship with digital technologies. Rather than having direct access to “objective” emotional responses, the “digitally mediated city” has already (re)organised the spatial and-temporal practices and discourses though which we come to see and experience ourselves [72]. The body is not directly accessible to science, and humility is needed in the adoption of biosensing technologies which make this promise. It is therefore be apposite for urban emotion researchers to draw on the insights of critical, feminist and participatory GIS approaches to design methodologies. Integrated methodologies which allow for multiple interpretations of the construct and causes of stress could enable analysis across different scales, across vectors of social difference, and advance knowledge about how stress is conceived and experienced in specific times and spaces.

## 5. Conclusions

Technological, engineering, architectural and ‘smart’ solutions to issues of urban mobility, commute and work stress are not neutral, and cannot necessarily fix complex social problems relating to the impacts of urban environments on emotional experience. Indeed, computer code can mediate our everyday experience of urban space, reproduce inequalities, silence marginalised groups, concentrate power and amplify social differences [73]. Acknowledging this requires more integration of objective and subjective emotions in urban emotion sensing methodologies than is commonplace. It needs careful attention to be paid to the concordance and dissonance between physiological, psychological, and experiential metrics and it necessitates a more nuanced understanding of the circular nature of the technologically-mediated contexts in which we become subjects, in which we interact, and in which we conduct research.

Future urban emotion sensing research therefore needs to more fully embrace the turn to social science and humanities accounts of emotional reflection and narrative. Even if we can now track emotional responses as people move through space, this kind of data doesn’t always lead to improved explanation and understanding of emotional experience. We are already witnessing how real-time emotions sensing places an economic value on “affective capture” and outsources analysis of our emotions to digital data experts [74]. This gives a false sense that the data ‘do not lie’, hiding the practices, stages and mechanisms of interpretation from view amid bold claims to know, track and manage urban emotions. Urban emotion researchers can thus help by developing interdisciplinary methodologies which value multiple understandings of stress and a diversity of epistemological standpoints. These complex form of methodological integration are far from easy, but they provide the basis for ensuring ethical principles of participation, voice and reflexivity have the potential to cultivate new forms of data citizenship.

## Figures and Tables

**Table 1:** Description of study participants.

Participant Details		Mean (SD)/*N* (%)		
		Birmingham	Salzburg	All
# Participants		22	9	31
Gender	Male	3 (14%)	8 (89%)	11 (35%)
	Female	19 (86%)	1 (11%)	20 (65%)
Age (years)		42.7 (11.3)	31.3 (8.9)	39.4 (11.7)
Marital Status	Cohabiting	7 (32%)	4 (50%)	11 (37%)
	Married	8 (36%)	3 (38%)	11 (37%)
	Single	7 (32%)	1 (13%)	8 (27%)
Education level	High School	6 (27%)	0 (0%)	6 (19%)
	Degree	11 (50%)	5 (56%)	16 (52%)
	Postgraduate	5 (23%)	4 (44%)	9 (29%)
Job Satisfaction	V. Satisfied	5 (23%)	5 (56%)	10 (32%)
	Satisfied	14 (64%)	4 (44%)	18 (58%)
	Neutral	2 (9%)	0 (0%)	2 (6%)
	Unsatisfied	1 (5%)	0 (0%)	1 (3%)
Duration of current employment	<2 years	5 (23%)	4 (44%)	9 (29%)
	2–5 years	7 (32%)	2 (22%)	9 (29%)
	6–10 years	4 (18%)	1 (11%)	5 (16%)
	>10 years	6 (27%)	2 (22%)	8 (26%)
Commute mode	Car	14 (48%)	0 (0%)	14 (35%)
	Cycle/Walk	0 (0%)	6 (55%)	6 (15%)
	Train/Bus	8 (28%)	3 (27%)	11 (28%)
	Mixed	7 (24%)	2 (18%)	9 (23%)
Commute duration	>15 min	0 (0%)	1 (11%)	1 (3%)
	15–30 min	10 (45%)	3 (33%)	13 (42%)
	30–45 min	5 (23%)	5 (56%)	10 (32%)
	45–60 min	4 (18%)	0 (0%)	4 (13%)
	>60 min	3 (14%)	0 (0%)	3 (10%)

**Table 2:** Psychometric survey results.

Psychometric Survey	Domains	Mean (SD)		
		Birmingham	Salzburg	All
Mean WHOQOL scores	Physical Health	65.2 (9.1)	74.5 (9.1)	67.9 (10.0)
	Psychological	69.0 (12.8)	80 (11.6)	72.2 (13.3)
	Social Relationships	78.0 (11.9)	77.8 (13.3)	77.8 (12.1)
	Environment	75.1 (10.0)	83.6(9.4)	77.5 (10.4)
	TOTAL	71.8 (7.3)	78.9 (7.5)	73.9 (7.9)
Mean PSS score		17.1 (5.0%)	11.5 (5.7)	15.5 (5.7)

**Table 3:** Descriptive statistics for PSS score by commute type.

Commute Type	*N*	Mean	SD
Cycle	3	9.67	7.02
Bus	2	17.50	2.12
Train	3	15.33	2.89
Car	12	19.17	4.82
Combined	8	13.75	4.13

**Table 4:** Descriptive statistics for WHOQOL scores by commute type.

Commute Type	*N*	Mean	SD
Cycle	3	83.00	10.82
Bus	2	69.75	3.18
Train	3	76.75	5.65
Car	12	69.23	6.48
Combined	8	74.84	7.16

**Table 5:** Coding framework and examples from qualitative interview data.

Thematic Code	Thematic Sub-Codes	Data Frequency	Summary of Data Examples
experimental study	(reflections of measurement of stress/emotions, the equipment and surveys, and of participating in the study)	59	Thought it would make me late for work; felt like the world’s ugliest FitBit/a tag for offenders (self-conscious); worries about how the wristband worked; lights kept flashing; not wanting to be honest with the food diary; worries that my data won’t be that useful; some people can hide their emotions; glad to get rid of the wristband; diaries are pretty accurate; emotions can be measured—sweat, pulse, going red; emotions are physical and independent from other bodily systems; easy, straightforward and quick processes; stress is a spectrum and depends on the person; I was aware of what might show on the wristband; too tight—flashes down my shoulder; wording of stress is ambiguous; the right words for my feelings weren’t there as options; some people are good at assessing their stress and others not at all; sometimes forgot to fill in diary every hour; can have different feelings during an hour; stress is a personal perception; I was just ticking the same thing each time; wasn’t sure what the questions were driving at; I don’t think stress and emotions can be measured just by people answering surveys; people may misinterpret questions or interpret words differently; maybe I do get stressed but just internalise it so answers may be different; everyone has their own threshold of stress; I like getting data about myself; I think I had it on upside down; I didn’t realise I was having such a crappy day until I filled that survey in; wanted to contribute to research; I never feel those tick boxes can capture anything very effectively
Health	-	9	
Home	activities at home physical environment at home relationships (at home) stressors at home	6 6 23 16	Partner suffering redundancy; caring for elderly parents; relationship ending; childcare worried; caring for children; partner in drug treatment programme; bereavement; wanting to move away closer to family; lack of time; lack of head space; tiredness; no free weekends; feeling of moaning at partner; ill-health of friends; lack of social support; own impatience; lack of time together as a family.
Journey description	calming or pleasant journey ideal journey nature spaces noticing, attention on journey people, relationships on journey stressors on journey traffic accident	21 37 23 35 34 43 11	Routes blocked; traffic jams; junctions onto main roads; worries about hitting cyclists; pinch points; bad drivers; badly planned junctions; lots of traffic; bins in the way; lack of parking; taxi drivers; lack of seats on train; funneling or rail passengers feeling like ‘cattle’; irritating passengers; noise; ice on roads/pavements; delays and cancellations; feeling of Russian roulette on the roads; children in the car; overcrowded trains/buses; buses that never come; feeling rammed in, pushing; being (late and) hungry; bad weather
Politics	-	10	
Self-tracking	-	5	
Stress	causes of stress coping with stress embodiment experience of stress meaning of stress Personality (effects on stress)	22 52 36 43 35 11	Tiredness; finances; personal relationships; politics and economic crisis; family illness/situations; big things; pre-existing anxieties; excessive workload; irritating or incompetent people; terrorism; Brexit; global wars; levels of poverty in society; hate crimes; lack of shared rules in society; looking after children; lack of social contact; heat; deadlines; traffic; lack of sleep; trying new things; things one can’t control Both negative and motivating, primeval response; damaging to health; feelings of anxiety; being out of control/a lack of control; headaches and physical symptoms; being in an unwanted position; having a lot on your plate; struggling to cope with a situation; interference in your life; increased heart rate; feeling squished (in time and space); time pressure; adrenaline; alert; on the knife edge; not thinking clearly; conflicting demands; (muscle) tension; feeling pushed out; irritation; wound up; anger; making irrational decisions; being overwhelmed; holding you back; palpitations; hair falling out; lack of concentration; joint pain; too much to do; pre-occupation with negative thoughts; lack of time; pressure
